# Could School Function Be an Adolescent Health Vital Sign? Associations Between Gradebook Data and Substance Use Among Middle School Students

**DOI:** 10.1111/josh.70261

**Published:** 2026-10-11

**Authors:** Rosemarie DiPentino, Benjamin P. L. Meza, Nicholas Jackson, Kulwant K. Dosanjh, Rebecca N. Dudovitz, Mitchell D. Wong

**Affiliations:** ^1^ David Geffen School of Medicine University of California Los Angeles California USA; ^2^ Department of Pediatrics University of Washington School of Medicine, Seattle Children's Hospital Seattle Washington USA; ^3^ Division of General Internal Medicine and Health Services Research, Department of Medicine David Geffen School of Medicine, University of California Los Angeles Los Angeles California USA; ^4^ Division of General Pediatrics, Department of Pediatrics and Children's Discovery and Innovation Institute David Geffen School of Medicine, University of California Los Angeles Los Angeles California USA

**Keywords:** academic performance, adolescence, adolescent substance use, alcohol use, cannabis use, risk factors

## Abstract

**Background:**

Identifying early substance use and at‐risk adolescents is a challenge for public health. The objective was to identify variables in digital school records that might distinguish between youth at high and low risk of adolescent substance use.

**Methods:**

We performed a secondary analysis of sixth‐grade students followed annually through eighth grade in Los Angeles, California. The composite outcome was self‐reported lifetime use of alcohol, cannabis, or other substances by eighth grade. Independent variables tested were parent‐reported GPA, transcript‐recorded GPA, and gradebook data including summative (quizzes and tests), formative (homework), and engagement (classroom behavior and participation) data. We evaluated models for discriminative ability.

**Results:**

Of the 234 students included in our sample, 51.7% were female, 82.1% were Latino, and 47 reported the composite outcome. Gradebook formative and engagement scores were most consistently associated with substance use. A model including gradebook engagement score and female sex demonstrated moderate ability to discriminate (*c*‐statistic = 0.79).

**Implications for School Health Policy, Practice, and Equity:**

Findings support the potential for digital gradebook data to serve as an inexpensive, readily available, and moderately accurate risk indicator of adolescent substance use.

**Conclusions:**

Schools, families, and health systems may leverage increasingly digitized school data to identify and support youth even where traditional modes of screening are limited

## 1 Introduction

Parents and caregivers, schools, healthcare professionals, and public health practitioners have a shared interest in the well‐being of adolescents, yet they face barriers to detecting substance use and other risky behaviors, which often begin during this sensitive developmental period. Adolescents are reluctant to disclose risky behaviors to adults, and those most likely to engage in risky behaviors are also more likely to have relational conflict and other barriers to disclosure with their caregivers and other adults [1]. Physicians encounter similar challenges when screening patients during routine physicals [2]. Moreover, any advantages of physician–patient confidentiality are offset by the infrequency of these visits, limited time to address multiple health issues during visits—let alone performing a validated screening for substance use—and the fact that only about one third of adolescents present annually for a well‐child visit [3, 4]. Therefore, there is a clear need for better, more timely ways to identify adolescents who are engaging in substance use and other risky health behaviors in order to connect them to appropriate interventions on and off school grounds.

A wealth of evidence in the literature indicates that poor academic performance and health risk behaviors are strongly related in a mutually reinforcing feedback loop [5, 6, 7]. Adolescents' use of alcohol and cannabis adversely affects students' grade point average (GPA), years of educational attainment, attendance, and standardized test scores [8, 9, 10, 11, 12, 13]. Poor school engagement also predicts subsequent delinquent behaviors [14]. A smaller subset of the literature has examined how these associations may be leveraged for risk prediction, although such studies almost exclusively use data from national surveys with self‐reported attitudes and behaviors, electronic health records, and general school‐level variables [15, 16, 17]. A paucity of research has examined highly granular academic performance data for this purpose. We hypothesized that academic performance data are correlated with the risk of substance use and thus could potentially serve as an early risk indicator of adolescent behavioral health problems, rhetorically analogous to a “vital sign.”

Vital signs—which conventionally include measures such as heart rate or blood pressure—are fundamental to clinical care. These metrics are inherently not specific to any one problem but rather serve as a sensitive indicator that an underlying problem may exist. An effective vital sign should also be easily and frequently measured to detect changes in health. Traditional measures of academic achievement do not fulfill these criteria of an effective vital sign because these data are not obtained continuously and lack variability. For example, though GPA can be monitored throughout the school year, changes will not be observed until the release of academic progress reports that typically occur only two to three times per year in most schools. Like other measures of academic achievement, homework completion is an established predictor of academic success but has the advantage of being recorded continuously [18, 19, 20]. In recent years, widespread adoption of digital gradebooks has made data on homework, classroom assignments, and classroom behaviors readily available to students, caregivers, and school administrators on a weekly, if not daily basis [21, 22]. Despite this ease of access, little is known about the relationship between gradebook data and health risk behaviors among adolescents.

Our aim was to address the question of whether gradebook data and GPA, markers of school engagement and academic achievement, may be used to discriminate students at high and low risk of alcohol and cannabis use in a population of middle school students from predominantly low‐income backgrounds in Los Angeles, California. Digital gradebook data may be divided into three domains: summative (quiz and test scores), formative (homework scores), and engagement (classroom behavior and participation scores). Given the established relationship between substance use and certain measures of academic achievement, along with evidence supporting homework completion as a reliable marker for overall academic success, we hypothesized that formative gradebook data may accurately discriminate risk of adolescent substance use. We further hypothesized that GPA data, due to its relative infrequency and limited variability, would have weaker discriminative ability.

## 2 Methods

### 2.1 Participants

We conducted a secondary analysis of data from a randomized controlled trial, the Linking Information and Families Together (LIFT) study (ClinicalTrials.gov Identifier: NCT02129153) [23]. The LIFT study was a teacher‐masked randomized controlled trial of 318 seventh graders in Los Angeles, California, during which parents were sent weekly text messages, telephone calls, or emails about their child's missed assignments, grades, and behavior, with results demonstrating substantial reductions in substance use initiation. Participants were recruited in sixth grade from four public middle schools in Los Angeles, California from May 2, 2014, to March 30, 2015, and data collection continued through eighth grade [24]. We selected these schools for their use of online gradebook systems, enrollment of youth from predominantly low‐income households, and their willingness to participate. Public schools in Los Angeles mirror public school systems across the United States of America in which the majority of students come from low‐income households and a substantial proportion (56% in 2023) do not graduate with the minimum requirements to be eligible for a state college or university [25, 26].

### 2.2 Procedures

Student participants completed a self‐administered computer‐assisted survey at three time points: seventh grade fall (baseline), seventh grade spring (Wave 1), and eighth grade spring (Wave 2). Students received $5 for each survey. Using measures from the Youth Risk Behavior Surveillance Survey [27], we asked each participant about lifetime use of (1) alcohol and (2) cannabis (“marijuana”), (3) inhalants, and (4) other drugs.

At baseline and follow‐up waves, we also conducted a telephone survey with one parent or guardian for each student participant. In addition to questions about parent–child relationships, parents were asked “What kind of grades does your child get in school?” and could respond with one of the following: mostly A's, mostly B's, mostly C's, or mostly D's (parent‐reported GPA). They received $20 for each survey they completed.

Academic performance for each student was obtained from academic transcripts (sixth to eighth grade) and the electronic gradebook (seventh to eighth grade). Transcripts contained GPA for each academic semester. The electronic gradebook is an online data management system where teachers report three domains of each student's performance: summative (quiz and test scores), formative (homework scores) and engagement (classroom behavior and participation scores). Gradebook domain scores (summative, formative and engagement) were averaged across each academic year. We also calculated a total gradebook score and a trajectory variable describing change from seventh to eighth grade. Total scores were categorized relative to the annual median and classified as low‐stable, worsening, improving, or high‐stable. Trajectory categories were designed to capture both baseline standing and change over time because students with minimal score change may represent either persistently low‐performing or persistently high‐performing groups. Additional details are provided in the online supplement.

### 2.3 Instrumentation

The primary outcome was a composite outcome of any lifetime use of alcohol or cannabis reported by eighth grade. We also asked about use of other illicit drugs, but the prevalence was near zero and thus not included in the outcome measure of substance use.

We tested two overarching categories of academic performance as independent variables at baseline: (1) transcript‐recorded and parent‐reported GPA and (2) electronic gradebook‐derived measures (i.e., summative, formative, engagement, and trajectory). Transcript‐recorded GPA was reported on a scale of 0.0–4.0 and is calculated by summing the number of grade points earned and dividing by the total number of credits attempted (> 3.5 = average A, 2.4–3.4 = average B, 1.5–2.4 = average C, < 1.5 = failing grade). Independent variables also included self‐reported female sex and Latino‐ethnicity.

### 2.4 Data Analysis

For this secondary data analysis, the trial data were accessed July 24, 2024, to February 6, 2025. Using descriptive statistics and visualizations, we analyzed each of our variables and their relationships to one another. We described patterns of missingness overall and by study site (school) and incorporated site into our analysis of missingness. For the primary analyses, we limited analyses to participants with complete data on sex, eighth‐grade substance use, GPA measures, and gradebook variables (complete‐case analysis). One school did not record engagement data and therefore engagement models were limited to a predefined subgroup analysis. Complete‐case analyses were used for primary model evaluation because cross‐validated AUC estimation is difficult to pool reliably across multiply imputed datasets in small samples.

To develop discriminative models, we first examined bivariate associations between each independent variable and our primary outcome. We used a combination of data‐driven variable selection based upon statistical significance (*p* < 0.05) as well as conceptually driven variable selection to create six prediction models: Full, GPA, Gradebook, Gradebook Formative, Gradebook Engagement, and Gradebook Change. Full, GPA, and Gradebook models were designed for comparison, thus variables were selected to be comprehensive at the expense of parsimony. Variable selection in the Gradebook Formative, Gradebook Engagement, and Gradebook Change models focused on isolating different forms of gradebook data, optimized parsimony, and minimized risk of overfitting. To test our hypothesis that adding a variable for GPA would not improve the models, these latter three models were tested with and without adding a variable for parental‐reported GPA in seventh grade as this was the GPA variable with the strongest bivariate association with the outcome. We assessed discriminative performance of our models by cross‐validation with the *k*‐fold cross‐validation method available in STATA using the *cvauroc* command for binary outcomes [28]. After modeling a logit model for the eighth grade lifetime substance use outcome, we iteratively bootstrapped five independent “test” samples from which an average c‐statistic and 95% confidence interval were obtained. Sensitivity‐specificity curves were produced using the *lsens* command and we calculated the sensitivity‐specificity pair that optimized each model's Youden index.

As a sensitivity analysis, we repeated all prespecified models using multiple imputation by chained equations (40 datasets) in R (mice package). Measures of model discrimination were pooled across imputations using Rubin's rules. Additional information regarding imputation procedures is provided in the supplement.

The University of California Los Angeles institutional review board and each participating school approved this study. A written parental informed consent and a written minor assent were completed for every participant.

## 3 Results

Of 318 adolescents who participated in the study, 289 students completed the final eighth grade survey (91% retention) with an average duration of follow‐up of 16 months (range 12.5–21.0 months). We excluded 55 participants because they were missing one or more required variables (i.e., sex, eighth grade substance use, transcript‐recorded or parent‐reported GPA for sixth to seventh grades). One school site did not record engagement gradebook data and therefore a subgroup analytic sample was created excluding those individuals for analyses that included engagement gradebook data. The complete (*N* = 234) and subgroup (*n* = 170) analytic sets are compared in Table 1. Of the entire study cohort, 51.7% of participants identified as female and 82.1% identified as Hispanic/Latino ethnicity; 85.0% of participants identified their race as White, 10.3% Black, 0.4% Asian or Pacific Islander, 2.6% Native American, and 1.3% more than one race. Approximately half of participants (53.8%) were randomized to the intervention group in the parent randomized controlled trial. Lifetime substance use in sixth grade was 13.7%, and intention to use substances was 18.8%. Caregivers were 87.1% female, 19.7% spoke primarily English, and 41.0% reported completing a high school diploma or more schooling. A total of 47 participants (14.8%) reported lifetime use of substances in eighth grade (22 cases among females and 25 cases among males). GPA, summative, and formative measures were strongly correlated, whereas engagement scores demonstrated only moderate correlations with other academic indicators (Table S1).

**TABLE 1:** Baseline sample characteristics, 2014–2016, Los Angeles, CA.

Variable	Complete sample (*N* = 234), *n* (%)	Subgroup^a^ sample (*N* = 170), *n* (%)
Female	121 (51.7%)	92 (54.1%)
Hispanic/Latino	192 (82.1%)	145 (85.3%)
Race		
White	199 (85.0%)	150 (88.2%)
Black/African American	24 (10.3%)	12 (7.1%)
Asian/Pacific Islander	1 (0.4%)	1 (0.6%)
Native American	6 (2.6%)	5 (2.9%)
More than one race	3 (1.3%)	2 (1.2%)
Intervention group	126 (53.8%)	95 (55.9%)
Average GPA (sixth grade)	2.882 (0.576)	2.967 (0.534)
Lifetime substance use (sixth grade)		
Any	32 (13.7%)	18 (10.6%)
Alcohol	14 (6.0%)	9 (5.3%)
Cannabis	11 (4.7%)	7 (4.1%)
Intention to use substances (sixth grade)		
Any	44 (18.8%)	26 (15.3%)
Alcohol	37 (15.8%)	23 (13.5%)
Cannabis	22 (9.4%)	13 (7.6%)
Parental characteristics		
Female	203 (87.1%)	145 (85.3%)
Primarily English‐speaking	46 (19.7%)	31 (18.3%)
High school diploma	96 (41.0%)	68 (40.0%)
Annual income		
< $15,000	101 (45.1%)	71 (43.3%)
$15–29,000	62 (27.7%)	44 (26.8%)
≥ $30,000	61 (27.2%)	49 (29.9%)

Abbreviation: GPA, grade point average.

^a^ Subgroup analysis (*n* = 170) excluded students for which no gradebook engagement data were available.

Testing unadjusted bivariate associations between independent variables and eighth grade substance use revealed significant associations with gradebook data (formative OR = 0.95, *p* = 0.025; engagement OR = 0.86, *p* < 0.001; low‐stable change OR = 1.95, *p* = 0.044; and high‐stable change OR = 0.46, *p* = 0.038), as well as parent‐reported GPA in seventh grade (OR = 0.65, *p* = 0.026) but no significant association was found between transcript‐recorded GPA in sixth or seventh grade and the primary outcome (Table 2).

**TABLE 2:** Bivariate associations between potential predictors and lifetime substance use by eighth grade, 2014–2016, Los Angeles, CA.

	Odds ratio	*p*	Lower 95% CI	Upper95% CI
Female	1.12	0.727	0.60	2.09
Latino ethnicity	0.66	0.265	0.32	1.37
Parent‐reported GPA				
Baseline	1.05	0.808	0.71	1.54
Wave 1	**0.65**	**0.026**	**0.44**	**0.95**
Transcript‐recorded GPA				
Sixth grade	0.88	0.662	0.49	1.58
Seventh grade	0.71	0.158	0.44	1.14
Gradebook scores				
Summative, seventh grade	0.97	0.069	0.94	1.00
Formative, seventh grade	**0.95**	**0.025**	**0.91**	**0.99**
Engagement, seventh grade^a^	**0.86**	**0.000**	**0.80**	**0.93**
Change: low‐stable	**1.95**	**0.044**	**1.02**	**3.72**
Change: worsening	1.46	0.407	0.59	3.61
Change: improving	0.62	0.447	0.18	2.14
Change: high‐stable	**0.46**	**0.038**	**0.22**	**0.96**

*Note:* Bold rows are significant to alpha < 0.05.

Abbreviations: CI, confidence interval; GPA, grade point average.

^a^ Subgroup analysis (*n* = 170) excluded students for which no gradebook engagement data were available.

In the multivariable‐adjusted models (Table 3), gradebook engagement score was the strongest and most consistent predictor of substance use in eighth grade (OR = 0.84, *p* < 0.001). Gradebook formative score and change in gradebook score from seventh to eighth grades were also significantly associated with lifetime substance use in eighth grade (OR = 0.95, *p* = 0.020; OR = 0.39, *p* = 0.020, respectively).

**TABLE 3:** Discriminative models of lifetime substance use in eighth grade, 2014–2016, Los Angeles, CA.

	Full^a^		GPA only		Parent‐reportedGPA		GB only^a^	
	OR	*p*	OR	*p*	OR	*p*	OR	*p*
Female	**3.91**	**0.028**	1.14	0.718	1.30	0.431	2.28	0.119
Parent‐reported GPA								
Baseline	0.88	0.797	1.69	0.087				
Wave 1	0.37	0.055	**0.52**	**0.047**	**0.63**	**0.020**		
Transcript‐recorded GPA								
Sixth grade	0.36	0.284	0.92	0.874				
Seventh grade	2.83	0.274	0.85	0.734				
Gradebook scores								
Summative	1.09	0.327					1.00	0.816
Formative	1.02	0.786					1.00	0.955
Engagement	**0.77**	**0.000**					**0.84**	**0.003**
Change: low‐stable	Ref.						Ref.	
Change: worsening	0.76	0.799					4.31	0.093
Change: improving	0.81	0.836					0.71	0.766
Change: high‐stable	1.43	0.736					0.75	0.774

	GBformative		GB formative and GPA		GBengagement^a^		GB engagement and GPA^a^		GBchange		GB change and GPA	
	OR	*p*	OR	*p*	OR	*p*	OR	*p*	OR	*p*	OR	*p*
Female	1.31	0.409	1.33	0.393	2.50	0.066	**2.83**	**0.042**	1.46	0.429	1.63	0.326
Parent‐reported GPA												
Sixth grade												
Seventh grade			0.76	0.259			0.73	0.353			0.53	0.096
Transcript‐recorded GPA												
Sixth grade												
Seventh grade												
Gradebook scores												
Summative												
Formative	**0.95**	**0.020**	0.97	0.210								
Engagement					**0.84**	**0.000**	**0.86**	**0.000**				
Change: low‐stable									Ref.		Ref.	
Change: worsening									1.59	0.482	2.41	0.223
Change: improving									0.39	0.387	0.45	0.470
Change: high‐stable									**0.17**	**0.008**	0.34	0.161

*Note:* Bold rows are significant to alpha < 0.05.

Abbreviations: GB, gradebook; GPA, grade point average; OR, odds ratio.

^a^ Subgroup analysis (*n* = 170) excluded students for which no gradebook engagement data were available.

Comparing the prognostic accuracy of six models (Table 4), we found that gradebook engagement data had the greatest prognostic accuracy and furthermore found that including GPA in the model provided little additional discriminatory value (*c*‐statistic = 0.80, cross‐validated AUC = 0.79, 95% CI [0.683, 0.871]; *c*‐statistic = 0.80, cross‐validated AUC = 0.74, 95% CI [0.614, 0.856], respectively). Sensitivity‐specificity curves are provided in the supplement along with sensitivity‐specificity estimates that optimize the model's Youden index (Figures S1–S10). Sensitivity analyses using multiple imputation found very similar c‐statistic estimates as the primary analyses (Table S2).

**TABLE 4:** Discriminatory accuracy of multivariable‐adjusted models of lifetime substance use in eighth grade, 2014–2016, Los Angeles, CA.

Model	C‐statistic	Cross‐validatedAUC	95% CI [lower, upper]
Full^a^	0.85	0.72	[0.543, 0.797]
GPA only	0.67	0.63	[0.484, 0.717]
Gradebook only^a^	0.81	0.70	[0.597, 0.816]
Parent‐reported GPA in seventh grade	0.61	0.59	[0.422, 0.621]
Gradebook formative	0.60	0.60	[0.435, 0.633]
Gradebook formative + GPA	0.61	0.61	[0.434, 0.637]
Gradebook engagement^a^	0.80	0.79	[0.683, 0.871]
Gradebook engagement + GPA^a^	0.80	0.74	[0.614, 0.856]
Gradebook change	0.70	0.68	[0.518, 0.777]
Gradebook change + GPA	0.75	0.73	[0.557, 0.801]

Abbreviations: CI, confidence interval; GPA, grade point average.

^a^ Subgroup analysis (*n* = 170) excluded students for which no gradebook engagement data were available.

## 4 Discussion

Substance use is a significant public health concern that frequently initiates during early adolescence. Yet caregivers, educators, healthcare providers, and public health practitioners currently lack accessible, objective tools to identify adolescents at risk of engaging in such behaviors, hindering early intervention efforts. As the first step in a transparent process of examining data sources for their predictive value, this study found that students with lower gradebook scores reported higher rates of lifetime substance use through eighth grade and that simple predictive models using aggregated gradebook data encouragingly demonstrated moderate predictive accuracy. These models performed favorably compared to GPA‐based predictive models of important academic outcomes such as dropout [29, 30].

In particular, engagement scores offered better discriminatory ability to identify students who would go on to endorse substance use a year later when compared to traditional measures of student academic achievement (transcript‐recorded GPA and parent‐reported GPA). The formative gradebook score was an average of teacher‐recorded homework completion and showed similar discrimination to transcript‐recorded GPA. Future studies may consider further investigating this comparison as formative gradebook data has the combined advantages of being continuously recorded throughout the school year and demonstrates superior granularity compared to GPA. The engagement gradebook score, which averaged scores for classroom participation and behavior, demonstrated greater discriminative accuracy after adjusting for student sex with marginal added benefit of extending the model to include parent‐reported GPA in seventh grade (*c*‐statistic = 0.79 and 0.81, respectively). Prior studies examining the association between school achievement and substance use have largely relied on report card data, that is, transcript‐recorded GPA [31]. Thus, we compared results from a model using transcript‐recorded GPA as its main predictor and found the variable to be considerably less predictive of lifetime substance use (*c*‐statistic 0.63). Parent‐reported GPA was more informative than transcript‐recorded GPA but when added to a model that included gradebook scores, GPA data did not significantly correlate with substance use and contributed little to the model's performance.

Our findings align with existing literature demonstrating a bidirectional association between substance use and both long term (e.g., high school graduation, college attendance) and short‐term (e.g., GPA, truancy) academic outcomes [5, 6, 12, 32, 33]. Studies using GPA to predict substance use find a consistent inverse relationship that is moderated by multiple factors including gender, misbehavior, and attitudes about school such as perception of school difficulty and connectedness [34, 35]. Similarly, studies investigating youth‐reported substance use have found that youth who endorse substance use that interferes with school functioning suffer from poorer health outcomes broadly [36, 37]. In contrast to the current study, many of these studies rely on student self‐report of GPA, and few if any studies have examined gradebook data generally or disaggregated such data into thematic domains such as summative, formative, and engagement. A number of studies have used specialized survey instruments to measure psychological predictors and found that low student emotional or behavioral engagement is associated with increased likelihood of risky behaviors [38].

Gradebook domains likely capture different aspects of student functioning relevant for hypothesis generation. Summative measures may be more closely tied to academic mastery, such as test taking skills and cumulative knowledge acquisition, whereas formative and engagement measures may be more sensitive to behavioral, organizational, relational, and environmental influences (e.g., home environment being conducive for studying, student‐teacher conflict). The stronger associations observed for formative and engagement scores suggest these measures may capture contextual and psychosocial processes more proximal to substance use risk. However, these mechanisms were not directly examined and should be investigated in future studies.

### 4.1 Implications for School Health Policy, Practice, and Equity

While the use of gradebook data might be considered a passive public health monitoring system [39], that is, using existing data, others have proposed alternative approaches including active systems. One such example, the Pediatric Symptom Checklist (PSC), is a 17‐ or 35‐item questionnaire designed for clinical settings and measures several domains of psychosocial well‐being including internalization, externalization, and attention [40]. The checklist has been described as a “vital sign” for psychosocial functioning that aids health practitioners in identifying youth who may benefit from further psychosocial assessment and services [40]. Despite its high degree of efficacy as a screening tool, this approach may not identify all adolescents in need of additional assessment. Because the PSC is primarily implemented in a clinical setting, its data are biased toward those who have more consistent access to health care. In the United States, as much as one third of adolescents may not receive an annual well visit [41]. Therefore, school‐based identification systems may be, particularly, advantageous for greater representativeness and earlier identification of hard‐to‐reach youth. Yet in an already stretched and overburdened public education system, active systems reliant on repeated implementation and student self‐reporting may be more resource intensive and consequently less practical when compared to passive tools. Use of gradebook data does not require additional effort beyond teachers' routine reporting or rely on student self‐report. Thus, a passive identification system based upon such data would be expected to have greater long‐term feasibility and sustainability. If sufficiently refined, a digital gradebook‐based system could potentially have minimal infrastructure or data collection requirements beyond existing school data systems, flexibly integrate different sources of data, and provide continuous monitoring in a setting attended by nearly all adolescents nationally.

While the use of gradebook data for health may provide potential advantages for achieving health equity, the incorporation of educational data into passive health monitoring systems presents unique challenges and ethical considerations. Among the challenges are that data not intended for health analytics should be carefully scrutinized, and educational data systems, designed for evaluative and administrative purposes, are no exception. Unlike a true vital sign, the collection of which should be standardized, calibrated, and harmonized to provide consistent meaning across settings, gradebook and related educational data can be messy and reflect more than underlying student characteristics. For example, scores may reflect differences in classroom management, disciplinary norms, grading culture, and implicit bias. Thus, biases may arise from contextual factors including the quality and consistency of data collection at multiple levels (i.e., teacher, school, district). In such messy settings, prediction modeling can amplify biases and cause unintended harm, particularly for socially marginalized groups [42]. Standards for transparency of the models themselves, monitoring differential performance across populations, and rectifying inequities are needed wherever predictive modeling is used and especially in the school context.

A number of ethical considerations also arise. Educational data collection is typically compulsory, thus placing the burden on institutions (e.g., schools, districts, third‐party vendors) to develop transparent mechanisms by which consent is obtained and safeguards to privacy and confidentiality are followed—particularly, when addressing potentially illicit behaviors. At a minimum, the ethical use of educational data is circumscribed by the Family Educational Rights and Privacy Act (FERPA), which emphasizes the importance of obtaining consent, limiting access, maintaining and documenting a clear intervention purpose, and avoiding the use of data for discipline, profiling, or surveillance [43]. Other municipal, state, and federal regulations may also apply depending on the nature of the data and intervention. Finally, systems for identifying at‐risk youth must be designed to enhance and not undermine social relationships between members of the community. Without forethought, protocols can displace relationships. The presence of any monitoring system should not substitute for adults investing in nurturing social connections and checking in with youth, regardless of quantifiable algorithmic parameters. Furthermore, if a student with a high‐risk profile is identified, such notification should trigger conversation about the root causes of health behaviors instead of rote punitive action. Given the potential stigma around being identified by an algorithm, procedures for utilizing these data must preserve student privacy and, ideally, be used as a platform to address existing stigma among youth and adults.

Schools have existing support systems that seek to identify vulnerable and struggling youth. In the most robust examples, multidisciplinary teams of teachers, counselors, nurses, psychologists, and administrators collect and analyze diverse forms of data such as parent and teacher concerns. A passive data monitoring system would constitute one source of data to be considered by such a team in the context of many other sources. Instead of a stand‐alone screening tool, such passive systems would most likely serve as a readily available contributor designed and integrated primarily to prevent high‐risk students from being missed by more selective and qualitative methods. The goal would be to facilitate earlier connection to resources and trusted adults rather than to label students or automate sensitive decision making (Figure S11).

### 4.2 Limitations

This study had several strengths and limitations. First, using data from a randomized trial, we were able to link rich gradebook data and a pressing health outcome, substance use. However, performing this secondary data analysis limited our sample size and the generalizability of our findings. While public middle schools in Los Angeles face many of the same challenges as public schools nationally, including prevalence of substance use, the results should be replicated in other populations and institutional settings.

Second, the study leveraged real‐life classroom data that avoids certain biases associated with self‐report of academic performance and lends support to the sustainability of future interventions using such data. Nevertheless, missingness is a real‐world problem which can impede prediction if missing data are informative. Most prominently, one of the four participating schools reported no gradebook engagement data and, as a result, was excluded from several components of the analysis given the risk of potential confounding at the school level. In our analysis, we chose a complete case approach; however, such an approach may lead to biased estimates. Similar findings from our multiply imputed data help ameliorate this concern. The absence of engagement data from one participating school also highlights a practical implementation challenge: schools differ substantially in what information is collected, digitized, and retained. The aforementioned school was part of a charter school network in which all schools had the digital infrastructure to collect gradebook data but differed in their reporting patterns. At the time of the study, this school had not developed a policy for digitally recording engagement data. Future studies should examine the feasibility of adapting predictive approaches across heterogeneous educational data systems. There is no national register detailing the types of data that schools record and digitize; therefore, any application of a similar method would need to be tailored to the setting in which it is implemented. Variation in grading practices, classroom culture and expectations, and the data systems themselves may influence the availability of data and the information they convey. Model performance will similarly vary, and local validation and calibration will likely be necessary.

The variables used here, including quizzes, tests, homework completion, and classroom participation and behaviors, are common domains of student assessment. This analysis used real‐world data in which teachers received no special data collection training. The relative stability of the engagement predictor after imputation (Table S2) despite having the greatest missingness may provide some support for the potential portability of the findings to other schools. Nevertheless, our study is limited in this respect, and a larger study with more schools and a greater diversity of data systems would allow a more precise analysis of data quality issues and administrative barriers to future predictive analytic tools.

Beyond replication, future research should explore how predictive tools developed in partnership with schools and communities can improve performance while remaining aligned with local priorities and values. Self‐reported lifetime substance use is a useful indicator of risky health behaviors, but future studies should examine the degree to which gradebook data may predict other aspects of whole child health including substance misuse [37] and substance use disorders, high‐risk sex behaviors, violence, bullying, and mental health disorders such as anxiety and depression. Results suggesting a similar predictive value for other adolescent problems would enhance the utility of gradebook data as a more broadly applicable predictive indicator. Future research should also validate these findings in rural, suburban, and socioeconomically diverse populations and determine whether combining gradebook measures with other routinely collected school health indicators improves discriminatory performance.

## 5 Conclusion

Considering that early substance use initiation is linked to other health risk behaviors [44], identifying teens at greatest risk is critical to early intervention and ensuring their future well‐being. While further study is necessary, the present study demonstrated the discriminative ability of different forms of academic data to correlate with adolescents reporting lifetime use of substances. Gradebook data was found to have higher predictive value when compared to traditional measures of academic performance including GPA. The findings represent an early step in a proof of concept to use gradebook data as a potential indicator, rhetorically analogous to an adolescent behavioral health “vital sign,” which may be particularly relevant for youth who do not routinely access the health care system. We use “vital sign” as a conceptual analog and not an established clinical construct. The findings provide encouraging evidence that gradebook engagement data may function as an early risk indicator, but additional validation across more diverse settings will be necessary before this terminology can be fully supported. Future studies can explore the clinical utility and validity of any screening tools developed from these findings. Adopting a simple tool that detects the most at‐risk students early on could allow schools, teachers, parents, and caregivers to intervene with targeted formal health assessments and appropriate early intervention. As school data becomes digitized and health and education systems become more integrated, future research should examine best practices for developing and implementing predictive analytic tools suitable for a health and well‐being ecosystem equipped to identify, support, and allocate important resources to youth in an efficient, ethical, and equitable way.

## Funding

This work was supported by the National Institutes of Health (KL2TR001882) and the Health Resources and Services Administration of the US Department of Health and Human Services (UA6MC32492), the Life Course Intervention Research Network.

## Ethics Statement

The University of California Los Angeles Institutional Review Board and each participating school approved this study.

## Consent

A written parental informed consent and a written minor assent were completed for every participant.

## Conflicts of Interest

The authors declare no conflicts of interest.

## Supporting information


**Data S1:** josh70261‐sup‐0001‐Supinfo.docx.
**Table S1:** Correlations between independent variables.
**Figure S1:** Sensitivity‐specificity plot: full model. Sensitivity and specificity across a range of probability thresholds are shown to illustrate the model's performance tradeoff: higher sensitivity optimizes true positives but comes at the cost of reduced specificity resulting in fewer true negatives. The Youden Index (*J* = sensitivity + specificity − 1) is one data‐driven method of identifying an optimal threshold that gives sensitivity and specificity equal weight: 95.5% sensitivity and 67.6% specificity (Youden Index = 0.63).
**Figure S2:** Sensitivity‐specificity plot: GPA model. Sensitivity and specificity across a range of probability thresholds are shown to illustrate the model's performance tradeoff: higher sensitivity optimizes true positives but comes at the cost of reduced specificity resulting in fewer true negatives. The Youden Index (*J* = sensitivity + specificity − 1) is one data‐driven method of identifying an optimal threshold that gives sensitivity and specificity equal weight: sensitivity 60.5% and specificity 68.7% (Youden Index = 0.29).
**Figure S3:** Sensitivity‐specificity plot: gradebook model. Sensitivity and specificity across a range of probability thresholds are shown to illustrate the model's performance tradeoff: higher sensitivity optimizes true positives but comes at the cost of reduced specificity resulting in fewer true negatives. The Youden Index (*J* = sensitivity + specificity − 1) is one data‐driven method of identifying an optimal threshold that gives sensitivity and specificity equal weight: sensitivity 81.8% and specificity 75.7% (Youden Index = 0.58).
**Figure S4:** Sensitivity‐specificity plot: parent‐reported GPA in seventh grade model. Sensitivity and specificity across a range of probability thresholds are shown to illustrate the model's performance tradeoff: higher sensitivity optimizes true positives but comes at the cost of reduced specificity resulting in fewer true negatives. The Youden Index (*J* = sensitivity + specificity − 1) is one data‐driven method of identifying an optimal threshold that gives sensitivity and specificity equal weight: sensitivity 59.6% and specificity 55.3% (Youden Index = 0.15).
**Figure S5:** Sensitivity‐specificity plot: gradebook formative model. Sensitivity and specificity across a range of probability thresholds are shown to illustrate the model's performance tradeoff: higher sensitivity optimizes true positives but comes at the cost of reduced specificity resulting in fewer true negatives. The Youden Index (*J* = sensitivity + specificity − 1) is one data‐driven method of identifying an optimal threshold that gives sensitivity and specificity equal weight: sensitivity 66.0% and specificity 52.0% (Youden Index = 0.18).
**Figure S6:** Sensitivity‐specificity plot: gradebook formative and GPA model. Sensitivity and specificity across a range of probability thresholds are shown to illustrate the model's performance tradeoff: higher sensitivity optimizes true positives but comes at the cost of reduced specificity resulting in fewer true negatives. The Youden Index (*J* = sensitivity + specificity − 1) is one data‐driven method of identifying an optimal threshold that gives sensitivity and specificity equal weight: sensitivity 80.9% and specificity 43.0% (Youden Index = 0.24).
**Figure S7:** Sensitivity‐specificity plot: gradebook engagement model. Sensitivity and specificity across a range of probability thresholds are shown to illustrate the model's performance tradeoff: higher sensitivity optimizes true positives but comes at the cost of reduced specificity resulting in fewer true negatives. The Youden Index (*J* = sensitivity + specificity − 1) is one data‐driven method of identifying an optimal threshold that gives sensitivity and specificity equal weight: sensitivity 96.0% and specificity 55.2% (Youden Index = 0.51).
**Figure S8:** Sensitivity‐specificity plot: gradebook engagement and GPA model. Sensitivity and specificity across a range of probability thresholds are shown to illustrate the model's performance tradeoff: higher sensitivity optimizes true positives but comes at the cost of reduced specificity resulting in fewer true negatives. The Youden Index (*J* = sensitivity + specificity − 1) is one data‐driven method of identifying an optimal threshold that gives sensitivity and specificity equal weight: sensitivity 88.0% and specificity 68.0% (Youden Index = 0.56).
**Figure S9:** Sensitivity‐specificity plot: gradebook change model. Sensitivity and specificity across a range of probability thresholds are shown to illustrate the model's performance tradeoff: higher sensitivity optimizes true positives but comes at the cost of reduced specificity, resulting in fewer true negatives. The Youden Index (*J* = sensitivity + specificity − 1) is one data‐driven method of identifying an optimal threshold that gives sensitivity and specificity equal weight: sensitivity 75.0% and specificity 47.0% (Youden Index = 0.22).
**Figure S10:** Sensitivity‐specificity plot: gradebook change and GPA model. Sensitivity and specificity across a range of probability thresholds are shown to illustrate the model's performance tradeoff: higher sensitivity optimizes true positives but comes at the cost of reduced specificity, resulting in fewer true negatives. The Youden Index (*J* = sensitivity + specificity − 1) is one data‐driven method of identifying an optimal threshold that gives sensitivity and specificity equal weight: sensitivity 65.9% and specificity 59.3% (Youden Index = 0.25).
**Table S2:** AUC of multivariable‐adjusted models of lifetime substance use in eighth grade using multiply imputed data, 2014–2016, Los Angeles, CA.
**Figure S11:** Conceptual framework for implementation of a school‐based passive data monitoring system. Gradebook‐derived risk indicators may support ongoing holistic assessment of student well‐being by identifying potential concerns that warrant further review. High‐risk scenarios may undergo multidisciplinary review, followed by private student outreach and additional information gathering, development of a multidisciplinary action plan, and connection to appropriate support services. This framework is illustrative and emphasizes supportive intervention rather than disciplinary action.

## Data Availability

The data that support the findings of this study are available on request from the corresponding author. The data are not publicly available due to privacy or ethical restrictions.
