# Supplementary material for: Could School Function Be an Adolescent Health Vital Sign? Associations Between Gradebook Data and Substance Use Among Middle School Students
Source: J Sch Health. 2026 Oct 11;96(10):e70261. doi: 10.1111/josh.70261 (PMC13654624; doi:10.1111/josh.70261)
Supplement: Supplementary file 1 [file JOSH-96-0-s001.docx]

**Article title**: Could school function be an adolescent health vital sign? Associations between gradebook data and substance use among middle school students

**Author names**: Rosemarie DiPentino, Benjamin P. L. Meza, Nicholas Jackson, Kulwant K. Dosanjh, Rebecca N. Dudovitz, Mitchell D. Wong

**Affiliation and email of corresponding author**: Benjamin P.L. Meza, UCLA Division of General Internal Medicine and Health Services Research, bpmeza@mednet.ucla.edu

Online Resources

# I. METHODS SUPPLEMENT

## Derivation of Academic Measures

Academic performance variables were derived from official school transcripts and electronic gradebook systems. Transcript-recorded GPA was obtained from school records and reported on a 0.0-4.0 scale. Parent-reported GPA was obtained from caregiver surveys and categorized as mostly A's, mostly B's, mostly C's, or mostly D's. Electronic gradebook systems recorded three broad domains of academic performance:

- **Summative scores:** quizzes, tests, and other cumulative assessments;
- **Formative scores:** homework and assignment completion;
- **Engagement scores:** classroom participation and behavior.

For each student, scores within each domain were averaged across the academic year to produce annual summative, formative, and engagement scores. A total gradebook score was calculated as the mean of the three gradebook domains. To examine changes in performance over time, total gradebook scores were categorized relative to the median score observed within each year. Students were then assigned to one of four trajectory categories between 7th and 8th grade:

- **Low-stable:** below the median in both years;
- **Worsening:** at or above the median in 7th grade and below the median in 8th grade;
- **Improving:** below the median in 7th grade and at or above the median in 8th grade;
- **High-stable:** at or above the median in both years.

Trajectory categories were used to capture both absolute level of performance and change over time. For example, students with little change in score could represent persistently low-performing or persistently high-performing groups despite having similar numeric change values.

## Missing data

Missing data were examined descriptively overall and by participating school. One participating school did not routinely record engagement-gradebook measures. Because engagement variables constituted a primary predictor of interest, analyses involving engagement scores were conducted as predefined subgroup analyses limited to schools in which such data were available.

The primary analyses used a complete-case approach requiring complete information on sex, outcome status, GPA variables, and gradebook measures included in each model. We chose the complete-case approach instead of multiple imputation for our primary analyses because of our preference to show cross-validated AUC estimates, which cannot be conducted on multiply imputed data without unstable performance and inflated variance due to the small sample sizes involved. To assess the potential impact of missingness on study findings, all primary analyses were repeated using multiple imputation as a sensitivity analysis (Table S2).

## Multiple imputation procedures

Sensitivity analyses were conducted using multiple imputation by chained equations implemented in R (mice package). Forty imputed datasets were generated. Missing values were imputed for outcome variables and covariates, including transcript-recorded GPA, parent-reported GPA, and gradebook domain scores. School site information was incorporated into the imputation process because missingness varied across participating schools. Derived measures, including total gradebook scores and gradebook trajectory indicators, were generated within each imputed dataset using passive imputation procedures. This approach ensured that derived variables remained consistent with their component variables while minimizing risks of mathematical incompatibility or collinearity. Within each imputed dataset, the prespecified logistic regression models were refit using the same predictor specifications as the primary analyses. Apparent model discrimination was evaluated using the c-statistic. Estimates were pooled across the 40 imputed datasets using Rubin's rules on the logit-transformed c-statistic scale and subsequently back-transformed for presentation. Results of the multiple-imputation analyses are presented in Table S6.

# II. Results Supplement

| Table S1. Correlations between independent variables. | | | | | | | | | | | | | |
| --- | --- | --- | --- | --- | --- | --- | --- | --- | --- | --- | --- | --- | --- |
|  | 1 | 2 | 3 | 4 | 5 | 6 | 7 | 8 | 9 | 10 | 11 | 12 | 13 |
| 1. Female | 1.00 |  |  |  |  |  |  |  |  |  |  |  |  |
| 2. Latinx | 0.03 | 1.00 |  |  |  |  |  |  |  |  |  |  |  |
| 3. Parent-reported GPA 6^th^ grade | 0.17 | 0.04 | 1.00 |  |  |  |  |  |  |  |  |  |  |
| 4. Parent-reported GPA 7^th^ grade | 0.10 | 0.11 | 0.50 | 1.00 |  |  |  |  |  |  |  |  |  |
| 5. Transcript-recorded GPA 6^th^ grade | 0.10 | 0.07 | 0.73 | 0.62 | 1.00 |  |  |  |  |  |  |  |  |
| 6. Transcript-recorded GPA 7^th^ grade | 0.08 | 0.13 | 0.52 | 0.74 | 0.77 | 1.00 |  |  |  |  |  |  |  |
| 7. Gradebook summative (baseline) | 0.07 | 0.11 | 0.61 | 0.70 | 0.80 | 0.82 | 1.00 |  |  |  |  |  |  |
| 8. Gradebook formative (baseline) | 0.06 | 0.10 | 0.43 | 0.69 | 0.70 | 0.86 | 0.80 | 1.00 |  |  |  |  |  |
| 9. Gradebook engagement* (baseline) | 0.22 | 0.26 | 0.24 | 0.51 | 0.44 | 0.70 | 0.53 | 0.62 | 1.00 |  |  |  |  |
| 10. Gradebook score: low-stable | -0.10 | -0.09 | -0.32 | -0.52 | -0.50 | -0.64 | -0.61 | -0.70 | -0.55 | 1.00 |  |  |  |
| 11. Gradebook score: worsening | -0.05 | 0.03 | -0.04 | -0.08 | -0.02 | -0.10 | 0.07 | 0.00 | -0.18 | -0.19 | 1.00 |  |  |
| 12. Gradebook score: improving | 0.02 | -0.09 | -0.09 | -0.11 | -0.25 | -0.13 | -0.27 | -0.17 | -0.01 | -0.22 | -0.12 | 1.00 |  |
| 13. Gradebook score: high-stable | 0.10 | 0.12 | 0.37 | 0.57 | 0.61 | 0.70 | 0.67 | 0.71 | 0.59 | -0.61 | -0.35 | -0.39 | 1.00 |
| *Subgroup analysis (n=170) excluded students for which no gradebook engagement data were available. | | | | | | | | | | | | | |

Figure S1. Sensitivity-specificity plot: Full Model

**Figure S1. Sensitivity-specificity plot: Full Model.** Sensitivity and specificity across a range of probability thresholds are shown to illustrate the model’s performance tradeoff: higher sensitivity optimizes true positives but comes at the cost of reduced specificity resulting in fewer true negatives. The Youden Index (J = sensitivity + specificity -1) is one data-driven method of identifying an optimal threshold that gives sensitivity and specificity equal weight: 95.5% sensitivity and 67.6% specificity (Youden Index = 0.63).

Figure S2. Sensitivity-specificity plot: GPA Model

**Figure S2. Sensitivity-specificity plot: GPA Model.** Sensitivity and specificity across a range of probability thresholds are shown to illustrate the model’s performance tradeoff: higher sensitivity optimizes true positives but comes at the cost of reduced specificity resulting in fewer true negatives. The Youden Index (J = sensitivity + specificity -1) is one data-driven method of identifying an optimal threshold that gives sensitivity and specificity equal weight: sensitivity 60.5% and specificity 68.7% (Youden Index = 0.29).

Figure S3. Sensitivity-specificity plot: Gradebook Model

**Figure S3. Sensitivity-specificity plot: Gradebook Model.** Sensitivity and specificity across a range of probability thresholds are shown to illustrate the model’s performance tradeoff: higher sensitivity optimizes true positives but comes at the cost of reduced specificity resulting in fewer true negatives. The Youden Index (J = sensitivity + specificity -1) is one data-driven method of identifying an optimal threshold that gives sensitivity and specificity equal weight: sensitivity 81.8% and specificity 75.7% (Youden Index = 0.58).

Figure S4. Sensitivity-specificity plot: Parent-reported GPA in 7^th^ Grade Model

**Figure S4. Sensitivity-specificity plot: Parent-reported GPA in 7th Grade Model.** Sensitivity and specificity across a range of probability thresholds are shown to illustrate the model’s performance tradeoff: higher sensitivity optimizes true positives but comes at the cost of reduced specificity resulting in fewer true negatives. The Youden Index (J = sensitivity + specificity -1) is one data-driven method of identifying an optimal threshold that gives sensitivity and specificity equal weight: sensitivity 59.6% and specificity 55.3% (Youden Index = 0.15).

Figure S5. Sensitivity-specificity plot: Gradebook Formative Model

**Figure S5. Sensitivity-specificity plot: Gradebook Formative Model.** Sensitivity and specificity across a range of probability thresholds are shown to illustrate the model’s performance tradeoff: higher sensitivity optimizes true positives but comes at the cost of reduced specificity resulting in fewer true negatives. The Youden Index (J = sensitivity + specificity -1) is one data-driven method of identifying an optimal threshold that gives sensitivity and specificity equal weight: sensitivity 66.0% and specificity 52.0% (Youden Index = 0.18).

Figure S6. Sensitivity-specificity plot: Gradebook Formative and GPA Model

**Figure S6. Sensitivity-specificity plot: Gradebook Formative and GPA Model.** Sensitivity and specificity across a range of probability thresholds are shown to illustrate the model’s performance tradeoff: higher sensitivity optimizes true positives but comes at the cost of reduced specificity resulting in fewer true negatives. The Youden Index (J = sensitivity + specificity -1) is one data-driven method of identifying an optimal threshold that gives sensitivity and specificity equal weight: sensitivity 80.9% and specificity 43.0% (Youden Index = 0.24).

Figure S7. Sensitivity-specificity plot: Gradebook Engagement Model

**Figure S7. Sensitivity-specificity plot: Gradebook Engagement Model.** Sensitivity and specificity across a range of probability thresholds are shown to illustrate the model’s performance tradeoff: higher sensitivity optimizes true positives but comes at the cost of reduced specificity resulting in fewer true negatives. The Youden Index (J = sensitivity + specificity -1) is one data-driven method of identifying an optimal threshold that gives sensitivity and specificity equal weight: sensitivity 96.0% and specificity 55.2% (Youden Index = 0.51).

Figure S8. Sensitivity-specificity plot: Gradebook Engagement and GPA Model

**Figure S8. Sensitivity-specificity plot: Gradebook Engagement and GPA Model.** Sensitivity and specificity across a range of probability thresholds are shown to illustrate the model’s performance tradeoff: higher sensitivity optimizes true positives but comes at the cost of reduced specificity resulting in fewer true negatives. The Youden Index (J = sensitivity + specificity -1) is one data-driven method of identifying an optimal threshold that gives sensitivity and specificity equal weight: sensitivity 88.0% and specificity 68.0% (Youden Index = 0.56).

Figure S9. Sensitivity-specificity plot: Gradebook Change Model

**Figure S9. Sensitivity-specificity plot: Gradebook Change Model.** Sensitivity and specificity across a range of probability thresholds are shown to illustrate the model’s performance tradeoff: higher sensitivity optimizes true positives but comes at the cost of reduced specificity, resulting in fewer true negatives. The Youden Index (J = sensitivity + specificity -1) is one data-driven method of identifying an optimal threshold that gives sensitivity and specificity equal weight: sensitivity 75.0% and specificity 47.0% (Youden Index = 0.22).

Figure S10. Sensitivity-specificity plot: Gradebook Change and GPA Model

**Figure S10. Sensitivity-specificity plot: Gradebook Change and GPA Model.** Sensitivity and specificity across a range of probability thresholds are shown to illustrate the model’s performance tradeoff: higher sensitivity optimizes true positives but comes at the cost of reduced specificity, resulting in fewer true negatives. The Youden Index (J = sensitivity + specificity -1) is one data-driven method of identifying an optimal threshold that gives sensitivity and specificity equal weight: sensitivity 65.9% and specificity 59.3% (Youden Index = 0.25).

| Table S2. AUC of multivariable-adjusted models of lifetime substance use in 8th grade using multiply imputed data, 2014-2016, Los Angeles, CA | |
| --- | --- |
| Model | C-statistic |
| Full ^a^ | 0.85 |
| GPA Only | 0.65 |
| Gradebook Only ^a^ | 0.80 |
| Parent-reported GPA in 7^th^ grade | 0.64 |
| Gradebook Formative | 0.61 |
| Gradebook Formative + GPA | 0.63 |
| Gradebook Engagement ^a^ | 0.80 |
| Gradebook Engagement + GPA ^a^ | 0.81 |
| Gradebook Change ^a^ | 0.70 |
| Gradebook Change + GPA ^a^ | 0.72 |
| Abbreviations: GPA – grade point average.  ^a^ Subgroup analysis (n=170) excluded students for which no gradebook engagement data were available. | |

# III. Discussion Supplement

**Figure S11. Conceptual framework for implementation of a school-based passive data monitoring system.**

|  | **Figure S11. Conceptual framework for implementation of a school-based passive data monitoring system.** Gradebook-derived risk indicators may support ongoing holistic assessment of student well-being by identifying potential concerns that warrant further review. High-risk scenarios may undergo multidisciplinary review, followed by private student outreach and additional information gathering, development of a multidisciplinary action plan, and connection to appropriate support services. This framework is illustrative and emphasizes supportive intervention rather than disciplinary action. |
| --- | --- |
